# *Lactiplantibacillus plantarum*-12 Alleviates Inflammation and Colon Cancer Symptoms in AOM/DSS-Treated Mice through Modulating the Intestinal Microbiome and Metabolome

**DOI:** 10.3390/nu14091916

**Published:** 2022-05-03

**Authors:** Fenglian Ma, Mengying Sun, Yinglong Song, Arong Wang, Shujuan Jiang, Fang Qian, Guangqing Mu, Yanfeng Tuo

**Affiliations:** 1 School of Food Science and Technology, Dalian Polytechnic University, Dalian 116034, China; fenglianma@163.com (F.M.); smy493006403@163.com (M.S.); sylong1016@163.com (Y.S.); wangarong2021@163.com (A.W.); jiangsj@dlpu.edu.cn (S.J.); qf09@163.com (F.Q.); 2 Dalian Probiotics Function Research Key Laboratory, Dalian Polytechnic University, Dalian 116034, China

**Keywords:** *Lactiplantibacillus*, metabolite, intestinal microbiota, colon cancer, caspase

## Abstract

In our previous research, *Lactiplantibacillus plantarum*-12 alleviated inflammation in dextran sodium sulfate (DSS)-induced mice by regulating intestinal microbiota and preventing colon shortening (*p* < 0.05). The purpose of the present study was to evaluate whether *L. plantarum*-12 could ameliorate the colon cancer symptoms of azoxymethane (AOM)/DSS-treated C57BL/6 mice. The results showed that *L. plantarum*-12 alleviated colonic shortening (from 7.43 ± 0.15 to 8.23 ± 0.25) and weight loss (from 25.92 ± 0.21 to 27.75 ± 0.88) in AOM/DSS-treated mice. *L. plantarum*-12 oral administration down-regulated pro-inflammatory factors TNF-α (from 350.41 ± 15.80 to 247.72 ± 21.91), IL-8 (from 322.19 ± 11.83 to 226.08 ± 22.06), and IL-1β (111.43 ± 8.14 to 56.90 ± 2.70) levels and up-regulated anti-inflammatory factor IL-10 (from 126.08 ± 24.92 to 275.89 ± 21.87) level of AOM/DSS-treated mice. *L. plantarum*-12 oral administration restored the intestinal microbiota dysbiosis of the AOM/DSS treated mice by up-regulating beneficial *Muribaculaceae, Lactobacillaceae,* and *Bifidobacteriaceae* levels and down-regulating pathogenic *Proteobacteria, Desulfovibrionaceae,* and *Erysipelotrichaceae* levels. As a result, the fecal metabolites of the AOM/DSS-treated mice were altered, including xanthosine, uridine, 3,4-methylenesebacic acid, 3-hydroxytetradecanedioic acid, 4-hydroxyhexanoylglycine, beta-leucine, and glycitein, by *L. plantarum*-12 oral administration. Furthermore, *L. plantarum*-12 oral administration significantly ameliorated the colon injury of the AOM/DSS-treated mice by enhancing colonic tight junction protein level and promoting tumor cells death via down-regulating PCNA (proliferating cell nuclear antigen) and up-regulating pro-apoptotic Bax. (*p* < 0.05). Taken together, *L. plantarum*-12 oral administration could ameliorate the colon cancer burden and inflammation of AOM-DSS-treated C57BL/6 mice through regulating the intestinal microbiota, manipulating fecal metabolites, enhancing colon barrier function, and inhibiting NF-κB signaling. These results suggest that *L. plantarum*-12 might be an excellent probiotic candidate for the prevention of colon cancer.

## 1. Introduction

Colon cancer is one of the most common cancers, which ranks third in incidence and second in mortality among all cancers [1]. Previous studies have proven that the pathogenesis of most cases of colon cancer is related to environmental factors, especially intestinal commensal bacteria, pathogens, and chronic enteritis [2], while few cases have a heredity factor [3]. In addition, the occurrence of colon cancer is closely related to chronic inflammation, and previous studies have shown that colitis patients with inflammatory bowel diseases (IBD) have an increasing risk of colon cancer [4].

The human gastrointestinal tract is inhabited by at least 100 trillion microbe cells, 10-fold the number of human cells [5]. The intestinal microbes of humans affect human life and health by participating in host metabolism and immune system regulation [6,7,8]. It has been evidenced that patients with colon cancer show an imbalance of intestinal microbiota with increased conditional pathogenic bacteria and harmful metabolites and decreased beneficial microbes [9,10,11]. It is reported that probiotics have the function of restoring the balance of intestinal microbiota and immunity, regulating intestinal metabolites, and relieving inflammation [12]. To the best of our knowledge, *Lactobacillus* ferments sugar to produce lactic acid, which lowers the pH of the intestinal tract, restrains the growth of pathogenic bacteria, promotes the abundance of beneficial bacteria, and competes with pathogenic bacteria for colonic epithelial binding sites. *Lactobacillus* is a major component of the intestinal microbiota and is usually used as a probiotic. A large number of studies have shown that *Lactobacillus* has an ameliorative effect on the disease symptoms of the host by rebalancing the intestinal microbiota, restoring colonic tight junction protein expression, and regulating inflammatory factors [13,14,15,16]. It was reported that prior supplementation of both *L. rhamnosus* GG and celecoxib reduced tumor burden and up-regulated the expression of pro-apoptotic Bax in 1,2-dimethylhydrazine-treated rats [17]. Gamallat et al. reported that *L. rhamnosus* GG reduced colon tumor incidence by inducing tumor cell apoptosis and inhibiting inflammation [18].

In our previous studies, the strain *L. plantarum*-12 was found to alleviate inflammation in DSS-induced mice by regulating intestinal microbiota, preventing colon shortening, enhancing the intestinal barrier function, and improving immunity [19]. In this study, the effects of *L. plantarum*-12 oral administration on intestinal microbiota and metabolites in colitis-associated colon-cancer-model mice was explored by omics analysis to reveal mechanism of alleviating the symptoms of colon cancer mice, which will hopefully provide a prevention and treatment approach to colon cancer.

## 2. Materials and Methods

### 2.1. Material and Reagents

Radioimmunoprecipitation assay (RIPA, R0010) with 1% phenylmethanesulfonyl fluoride (PMSF) buffer was purchased from Solarbio Life Science (Beijing, China). The following primary antibodies were used for the Western blotting of colon tissue homogenate: Claudin-1(A11530) antibody (abclonal, Wuhan, Hubei, China), phosphorylated p38 (p-p38, 4511) and phosphorylated NF-κB (p-p65, 3033) antibodies (Cell Signaling, Danvers, MA, USA), Bax (AF0057), Inhibitor kappa B-α (IκB-α, AF1282), NF-κB (p65, AF0246), PCNA (AF1363), and β-actin (AF5003) antibodies (Beyotime Institute of Biotechnology, Shanghai, China).

### 2.2. Culture of L. plantarum-12

*L. plantarum*-12 was deposited at the Dalian Probiotics Function Research Key Laboratory, Dalian polytechnic university, in Dalian, China. *L. plantarum*-12 was sub-cultured in MRS medium with the initial pH 6.4 (Land Bridge Biotechnology, Beijing, China) at 37 °C for 20 h without anaerobic conditions. The pellets were collected by centrifugation at 3000× *g* for 10 min at 4 °C. The pellets were washed two times with sterile saline. Then, the pellets were re-suspended in sterile saline. Finally, the concentration of the suspension was adjusted to 2 × 10^7^ CFU mL^−1^ and 2 × 10^9^ CFU mL^−1^ for oral supplement, respectively.

### 2.3. Animals and Experimental Design

**Figure 1:**
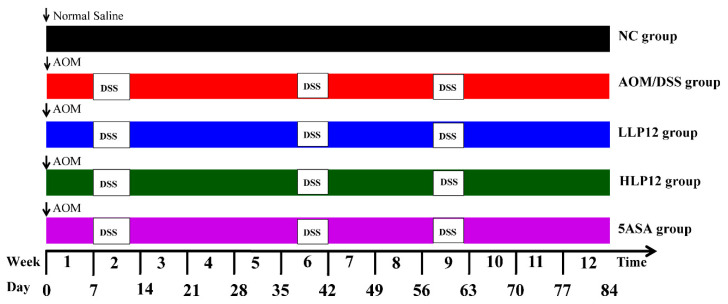
Experimental design of AOM/DSS-treated colon cancer models in the C57BL/6 mice (*L. plantarum*-12/5ASA, intragastric administration; AOM, intraperitoneal injection; DSS, administered by drinking water).

To explore the anti-tumor effects of *L. plantarum*-12, an AOM/DSS colitis-associated colon cancer model was used and slightly modified according to the method reported by Neufert [20]. All animal experiments were carried out according to the Guidelines of Experimental Animal Ethics Committee of Dalian Polytechnic University (SYXK2017-0005). Seventy-five 6-week-old male C57BL/6 mice were obtained from Liaoning Changsheng Biotechnology Co., Ltd., Benxi, China. Mice were kept under controlled environmental conditions at 22 ± 2 °C and relative humidity of 50% ± 10% with 12 h light/dark cycle with free access to water and food at the Animal Center of Dalian Polytechnic University, China. After acclimation for 2 weeks, all the mice were randomly assigned into five groups (*n* = 15 per group). As shown in Figure 1, (A) NC group: normal control with gavage daily normal saline; (B) AOM/DSS group: AOM/DSS with gavage daily normal saline; (C) LLP12 group: AOM/DSS plus gavage daily 250 μL of 2 × 10^7^ CFU mL^−1^ *L. plantarum*-12 suspension; (D) HLP12 group: AOM/DSS plus gavage daily 250 μL of 2 × 10^9^ CFU mL^−1^ *L. plantarum*-12 suspension; (E) 5-aminosalicylic acid (5ASA) group: AOM/DSS plus gavage daily 75 mg kg^−1^ body weight 5ASA. NC group received an injection of sterile saline, whereas the rest of the groups received an intraperitoneal injection of azoxymethane (AOM, Sigma Chemical Co., St. Louis, MO, USA) solution at a doge of 12.5 mg kg^−1^ body weight at the beginning of the experiment (day 0). NC group was administered drinking water, whereas other groups were administered drinking water containing 2.5% dextran sulfate sodium salt (DSS, MW: 40,000 Da, MP Biomedicals, Santa Ana, USA) for 5 days at 2, 6, and 9 weeks, respectively. The live body weight was recorded once a week throughout the experiment. At the end of the experiment, the mice feces were collected, and blood samples were attained from mice eyeballs. The serum was separated at 3000× *g* for 10 min at 4 °C and stored at −80 °C. The mice were euthanized by cervical dislocation at week 12. The colon, spleen, thymus, and liver were obtained and measured for weight.

### 2.4. Histological Analysis and Biochemical Analysis

Fresh colon tissues of the C57BL/6 mice were quickly taken and fixed with 4% paraformaldehyde for 48 h. After dehydrating with gradient ethanol and treating with paraffin wax, the colon tissues were sectioned into 4 μm slices. The sections were dewaxed with xylene and stained with hematoxylin and eosin. The images were acquired by an Olympus microscope (Olympus Optical Co., Ltd., Beijing, China).

The levels of tumor necrosis factor-α (TNF-α), interleukin-10 (IL-10), interleukin-8 (IL-8), and interleukin-1 beta (IL-1β) in the mice serum were tested using the commercial ELISA kits based on submitted instructions (Nanjing Jiancheng Bioengineering Institute, Nanjing, China).

### 2.5. Western Blotting Analysis

The lysis buffer (Solarbio Life Science, Beijing, China) was composed of RIPA and PMSF solution at a ratio of 99:1, which was used to extract protein of colon tissue samples of the C57BL/6 mice, following the instructions. The levels of protein were tested by the bicinchoninic acid (BCA) protein assay kit (Solarbio Life Science, China). Equivalent proteins were electrophoresed via 12% SDS-PAGE, and afterwards, the proteins were transferred onto polyvinylidene fluoride (PVDF) membranes (Millipore, Darmstast, Germany). The membranes were blocked with Tris-buffered saline Tween 20 (TBST) containing 5% skim milk at room temperature for 1 h. Furthermore, the membranes were incubated with appropriate primary antibodies (1:1000) at 4 °C overnight. After washing five times, the membranes were incubated with horseradish peroxidase (HRP)-conjugated secondary antibodies (1:1000) for 1 h at room temperature. After washing five times, BeyoECL star Kit (Beyotime Institute of Biotechnology) was then used to detect protein bands according to providing directions. A chemiluminescence imaging system was used to visualize the protein by an image scanner (Azure C300, Azure Biosystems, Dublin, CA, USA), which was quantified via the NIH Image J software (National Institutes of Health, Bethesda, MD) and normalized to β-actin.

### 2.6. Intestinal Microbiota Analysis

Twenty fecal samples of the C57BL/6 mice were randomly selected from the NC, AOM/DSS, HLP12, and 5ASA groups (five samples per group) for the 16S rDNA sequencing analysis. Microbial DNA was extracted from mice feces using the E.Z.N.A.^®^ soil DNA Kit based on the protocol [16]. The DNA concentration was determined by NanoDrop 2000 UV-vis spectrophotometer. The V3-V4 region of the 16S rRNA gene was amplified using universal primer 338F (5′-ACTCCTACGGGAGGCAGCAG-3′) and 806R (5′-GGACTACHVGGGTWTCTAAT-3′) by an ABI GeneAmp^®^ 9700 PCR thermocycler (ABI, CA, USA). Subsequently, the amplified DNA fragments were checked on 2% agarose gel electrophoresis. Based on the standard protocols of Majorbio Bio-Pharm Technology Co., Ltd. (Shanghai, China), equimolar concentrations of purified amplicons were paired-end sequenced on an Illumina MiSeq platform (Illumina, San Diego, CA, USA).

The raw 16S rRNA gene sequencing reads were demultiplexed, quality-filtered by Trimmomatic, and merged by FLASH version 1.2.7. Operational taxonomic units (OTUs) were clustered with 97% similarity cutoff using UPARSE version 7.1, and chimeric sequences were identified and removed using UCHIME. The taxonomy of each OTU representative sequence was analyzed by RDP Classifier version 2.2 based on the Silva database. Alpha diversity (Shannon index and Chao index) of intestinal microbiota based on the OTU level was calculated by R software package. Beta diversity (Principal coordinates analysis, PCoA) plot was produced with the ANOSIM. Bar plot of the community abundance distribution at the phylum level and family level was analyzed using the R software package. Significant differences in differential species between the two groups were determined by two-tailed Wilcoxon rank-sum test. The linear discriminant analysis (LDA) effect size (LEfSe) was used to identify the bacterial biomarkers from phylum to genus level between groups, and LDA score >2.0.

### 2.7. Fecal Metabolomic Analysis

Twenty fecal samples of the C57BL/6 mice were randomly selected from the four groups (five samples per group) for mice fecal metabolites analysis. The analysis of mice fecal metabolites by LC-MS was performed as previously described [21] but slightly changed. Briefly, feces sample of the C57BL/6 mice (50 mg) were collected in 2 mL thick centrifuge tube, and steel ball was added. Fecal metabolites were extracted by 400 µL extract (methanol: water = 4:1) and 20 µL L-2- Chloro-phenylalanine (0.3 mg/mL) as the internal standard. Metabolites were checked by UPLC-Triple TOF system (AB SCIEX, Framingham, MA, USA) equipped with a HSS T3 column (100 mm × 2.1 mm id, 1.8 µm; Waters, Milford, CT, USA). In the process of instrumental analysis, a quality control (QC) sample was inserted in every 7 analysis samples to examine the repeatability of the entire analysis process. Progenesis QI (Waters, Milford, USA) was used for raw data preprocessing. The preprocessing results generated a data matrix that consisted of the retention time, mass-to-charge ratio values, and peak intensity. Data matrix was used to remove the missing value with 80% rule. After filtering, minimum metabolite values were imputed for specific samples in which the metabolite levels fell below the lower limit of quantitation, and all metabolic features were normalized by sum. Metabolic features for which the relative standard deviation (RSD) of QC > 30% were discarded. Following normalization procedures and imputation, statistical analysis was performed on log-transformed data to identify significant differences in metabolite levels between comparable groups. Finally, the data matrix was obtained for subsequent analysis. A principal component analysis (PCA) was performed to visualize metabolic alterations among QC and other groups. Differential metabolite analysis between groups was determined by two-tailed Wilcox’ test (Unpaired). KEGG pathway-enrichment analysis was performed by Python software package scipy. stats. The results were analyzed on the free online platform of Majorbio Cloud Platform (www.majorbio.com, accessed on 15 April 2022).

### 2.8. Statistical Analysis

Experiment data are presented as mean ± SD. Data were analyzed by one-way ANOVA using SPSS Statistics version 20.0 (IBM, Chicago, IL, USA). The results were considered statistically significant if *p* < 0.05. Spearman correlation heatmap was performed with the R software.

## 3. Results

### 3.1. Effect of L. plantarum-12 Oral Administration on the Body Weight and Colon Length of the AOM/DSS-Treated C57BL/6 Mice

**Figure 2:**
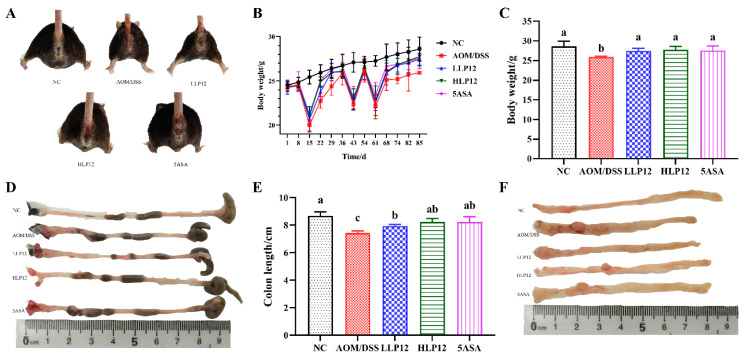
Effect of *L. plantarum*-12 oral administration on body weight and colon length of the AOM/DSS-treated C57BL/6 mice (*n* = 6). (**A**) Mice morphology, (**B**) mice body weight, (**C**) mice body weight on the 85th day, (**D**) colon morphology, (**E**) colon length of the mice, and (**F**) mice terminal colon morphologies under different treatments. The data with different lowercase letters are significantly different (*p* < 0.05).

At the beginning of the experimental period, the body weights of the C57BL/6 mice in the five groups were about 24 g (Figure 2B). When the mice drank 2.5% DSS water in the 2nd, 6th and 9th weeks, the body weights of the mice significantly decreased. When they drank purified water, the body weights of the mice recovered. As shown in Figure 2C, on the 85th day, the body weights of the mice in LLP12 and HLP12 groups were obviously higher than that of the mice in AOM/DSS group (*p* < 0.05). Except for the mice in NC group, the mice in the rest of the groups showed ulcers and blood in the anus (Figure 2A). As shown in Figure 2D,E, the colon length of the mice in AOM/DSS group was obviously shorter than that of the NC group (*p* < 0.05). However, the colon length of the mice in the LLP12, HLP12, and 5ASA groups recovered compared with that in the AOM/DSS group (*p* < 0.05). A large number of tumors were enriched in the distal colon of the mice in the AOM/DSS group, whereas the colon tumor number of the mice in the *L. plantarum*-12 oral administration groups decreased (Figure 2F).

### 3.2. Effect of L. plantarum-12 Oral Administration on Serum Inflammatory Cytokine and Organ Index of the AOM/DSS-Treated C57BL/6 Mice

**Figure 3:**
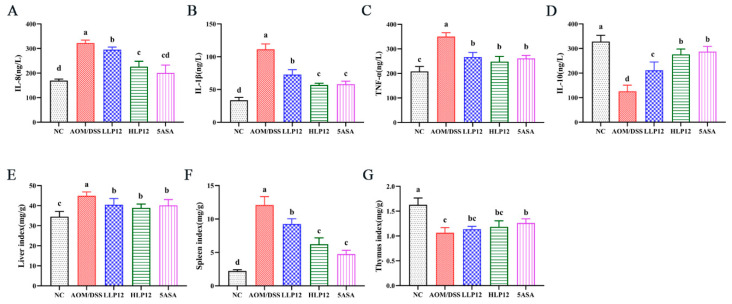
Effect of *L. plantarum*-12 oral administration on inflammatory cytokine and organ index of the AOM/DSS-treated C57BL/6 mice (*n* = 4). (**A**) IL-8, (**B**) IL-1β, (**C**) TNF-α, (**D**) IL-10, (**E**) liver index, (**F**) spleen index, and (**G**) thymus Index. The data with different lowercase letters are significantly different (*p* < 0.05).

As shown in Figure 3A–C, *L. plantarum*-12 oral administration was sufficient to remarkedly suppress the upregulation of pro-inflammatory factors, including IL-8, IL-1β, and TNF-α of the AOM/DSS-treated C57BL/6 mice. In addition, *L. plantarum*-12 oral administration reversed the IL-10 downregulation of the AOM/DSS-treated C57BL/6 mice (Figure 3D). Compared with NC group, the liver index and spleen index of the mice in AOM/DSS group were obviously higher (*p* < 0.05), as shown in Figure 3E,F. Meanwhile, the oral administration of *L. plantarum*-12 could significantly ameliorate the increase of the liver index and spleen index of the mice in the LLP12, HLP12, and 5ASA groups. The thymus index was decreased in the AOM/DSS group relative to NC group, whereas supplementation with *L. plantarum*-12 increased thymus index in the LLP12 and HLP12 groups, with no significant difference (Figure 3G).

### 3.3. Effect of L. plantarum-12 Oral Administration on Gut Barrier Function of the AOM/DSS-Treated C57BL/6 Mice

**Figure 4:**
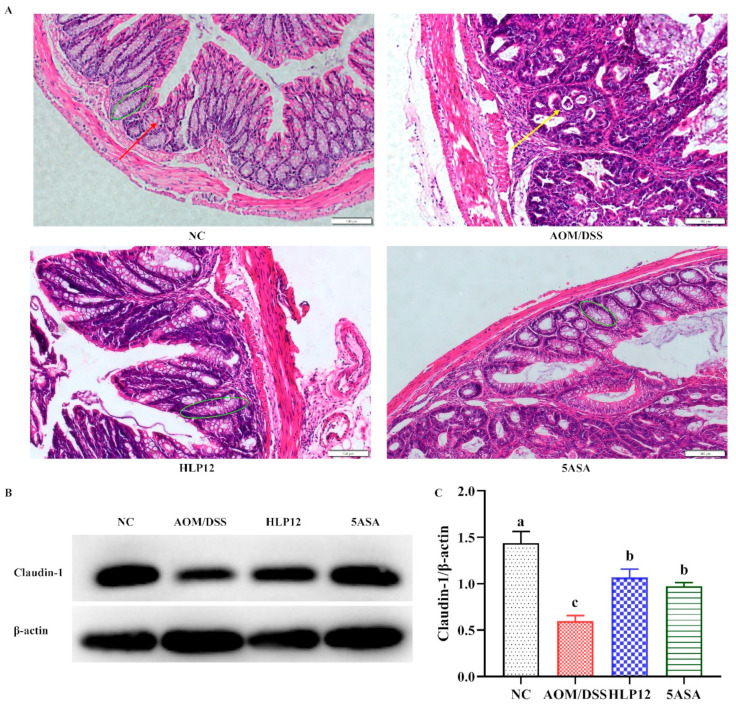
Effect of *L. plantarum*-12 oral administration on intestinal barrier function of the AOM/DSS-treated C57BL/6 mice. (**A**) Colon histological observation (Magnification: 200); the green oval represents the colonic crypt. The red arrow represents goblet cells. The yellow arrow represents adenomas. (**B**) The colon protein expression of Claudin-1 was determined by Western blotting, and (**C**) β-actin acted as control for the protein blots (*n* = 4). The data with different lowercase letters are significantly different (*p* < 0.05).

As shown in Figure 4A, compared with NC group, colon goblet cells and crypt structure of the C57BL/6 mice in AOM/DSS group were changed, whereas supplementation with *L. plantarum-*12 could recover crypt structure and increase the goblet cells. Then, the effect of supplementation with *L. plantarum*-12 on the expression of colon tight junction protein Claudin-1 was further detected via Western blotting. As shown in Figure 4B,C and Figure S1, *L. plantarum*-12 oral administration upregulated the protein expression of Claudin-1 (*p* < 0.05) in the colons of AOM/DSS-treated C57BL/6 mice.

### 3.4. Effect of L. plantarum-12 Oral Administration on the Protein Expression Related to p38 MAPK and NF-κB Signaling Pathways of the AOM/DSS-Treated C57BL/6 Mice

**Figure 5:**
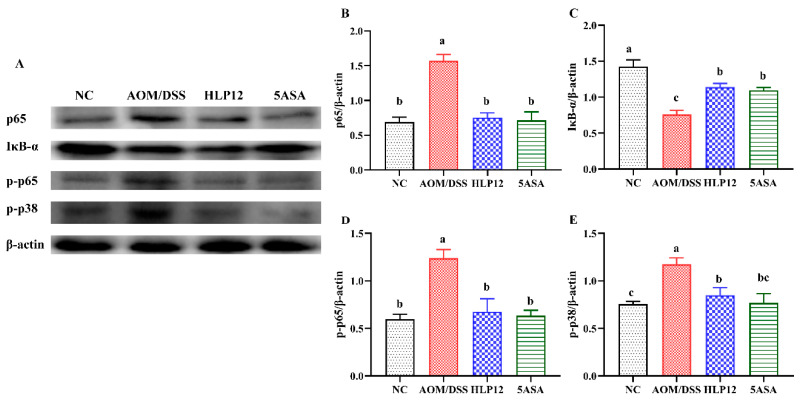
Effect of *L. plantarum*-12 oral administration on the protein expression related to p38 MAPK and NF-κB signaling pathways of the AOM/DSS-treated C57BL/6 mice. (**A**–**E**) p65, I*κ*B-α, p-p65, and p-p38 in the colon of AOM/DSS-treated mice were assessed by Western blotting (*n* = 4). The data with different lowercase letters are significantly different (*p* < 0.05).

To examine the mechanism whereby *L. plantarum*-12 was able to alleviate AOM/DSS-induced colon cancer of the C57BL/6 mice, the activation of p38 MAPK and NF-κB-signaling-pathways-associated proteins in the colon of the mice was assessed, as shown in Figure 5 and Figure S1. The results revealed that *L. plantarum*-12 oral administration was sufficient to suppress the phosphorylation of p38 MAPK (p-p38) of the AOM/DSS-treated C57BL/6 mice (Figure 5E). In addition, p65 (Figure 5B) and p-p65 (Figure 5D) expression levels were increased in AOM/DSS group, whereas *L. plantarum*-12 oral administration decreased the expression. Compared with NC group, IκB-α level was decreased in the AOM/DSS group, while *L. plantarum*-12 oral administration could improve the IκB-α level in the HLP12 group (*p* < 0.05), as shown in Figure 5C. As such, *L. plantarum*-12 oral administration was able to alleviate AOM/DSS-induced colon cancer via inhibiting the expression of p65, p-p65, and p-p38 phosphorylation in colon.

### 3.5. Effect of L. plantarum-12 Oral Administration on the Expression of Apoptosis-Related Proteins of the AOM/DSS-Treated C57BL/6 Mice

**Figure 6:**
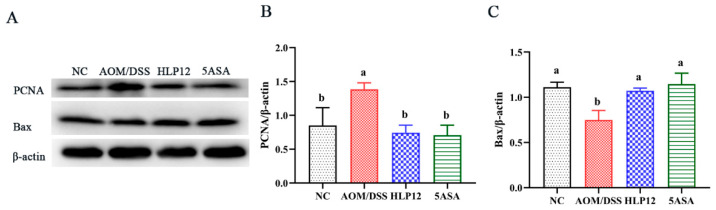
Effect of *L. plantarum*-12 oral administration on the expression of apoptosis-related proteins of the AOM/DSS-treated C57BL/6 mice. (**A**–**C**) PCNA and Bax in the colon of AOM/DSS-treated mice were assessed by Western blotting (*n* = 4). The data with different lowercase letters are significantly different (*p* < 0.05).

To elucidate the mechanism of tumor apoptosis, the Bax and PCNA proteins expression of the C57BL/6 mice colon were measured by Western blotting. AOM/DSS challenge increased the expression level of PCNA in the mice colon (*p* < 0.05); however, *L. plantarum*-12 oral administration reduced the expression of colonic PCNA in AOM/DSS-challenged mice (*p* < 0.05), as shown in Figure 6B and Figure S1. Furthermore, *L. plantarum*-12 oral administration significantly (*p* < 0.05) up-regulated the pro-apoptotic Bax expression compared with AOM/DSS group (Figure 6C and Figure S1). These results suggested that the ameliorating effect of *L. plantarum*-12 oral administration on colon cancer of the mice treated by AOM/DSS may be achieved by down-regulating PCNA and up-regulating the Bax, thereby promoting colon cancer cells apoptosis.

### 3.6. Effect of L. plantarum-12 Oral Administration on the Intestinal Microbiome of the AOM/DSS-Treated C57BL/6 Mice

**Figure 7:**
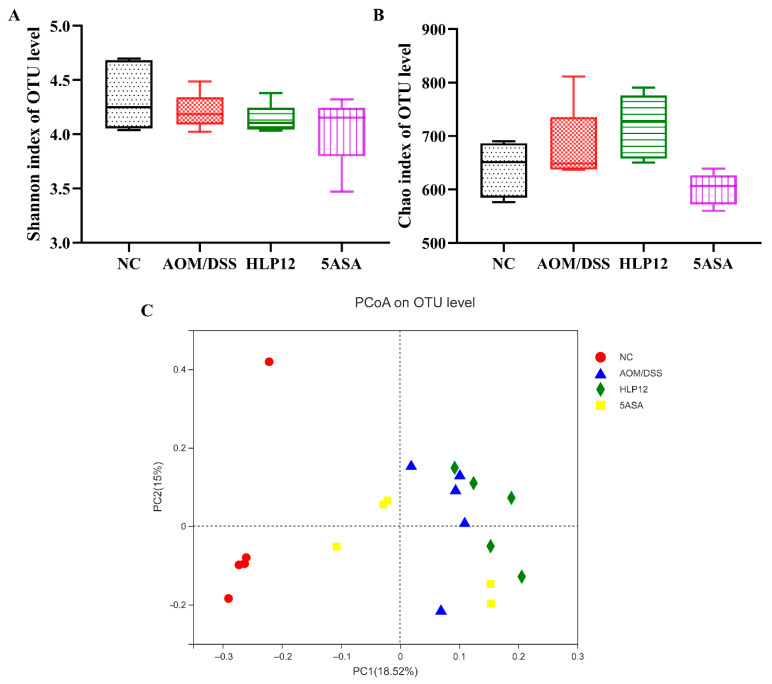
Effect of *L. plantarum*-12 oral administration on intestinal microbiota composition of the AOM/DSS-treated C57BL/6 mice (*n* = 5). (**A**) Shannon index of identified OTUs, (**B**) Chao index of identified OTUs, and (**C**) PCoA analysis.

We next employed a 16S rRNA gene high-throughput sequencing approach to evaluate the intestinal microbiota of the C57BL/6 mice in each group. Analysis of the α diversity index is displayed in Figure 7A,B. Shannon and Chao indexes among the NC, AOM/DSS, HLP12, and 5ASA groups have no significant difference. Furthermore, an obvious difference was observed in the PCoA between the NC and AOM/DSS groups (Figure 7C). It showed that the intestinal microbiota of mice in the NC and AOM/DSS groups were significantly changed, but no obvious changes were observed between the HLP12 and AOM/DSS groups (Figure 7C).

**Figure 8:**
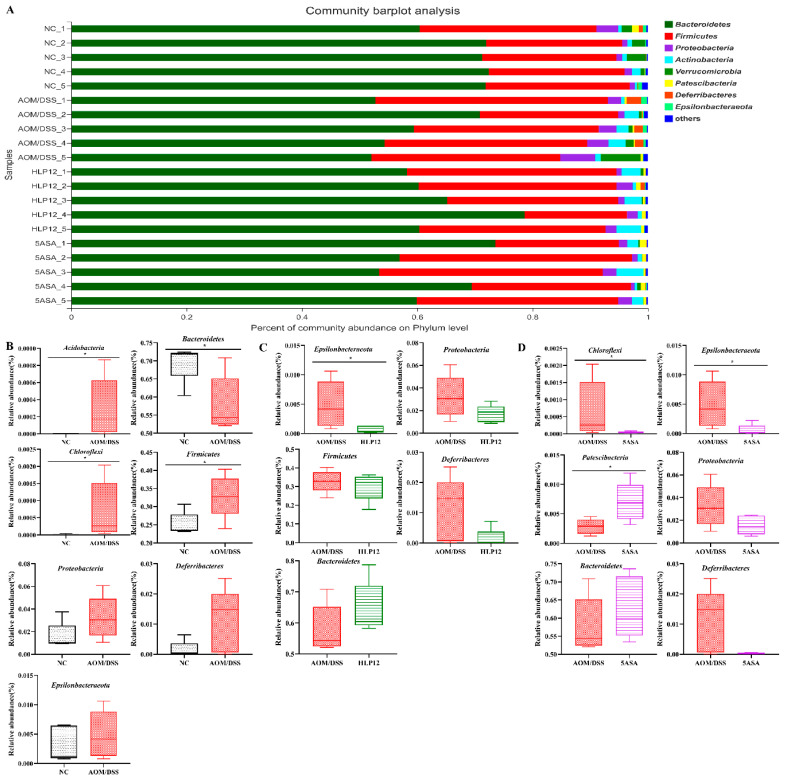
Effect of *L. plantarum*-12 oral administration on intestinal microbiota at the phylum level of AOM/DSS-treated mice (*n* = 5). (**A**) Bar picture, (**B**) NC vs. AOM/DSS species difference analysis, (**C**) AOM/DSS vs. HLP12 species difference analysis, and (**D**) AOM/DSS vs. 5ASA species difference analysis. * *p* < 0.05.

**Figure 9:**
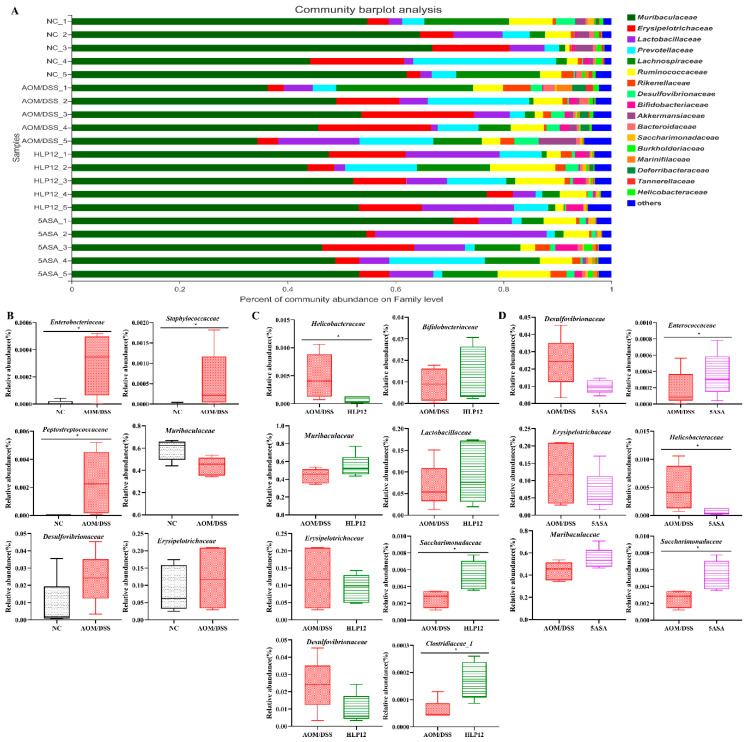
Effect of *L. plantarum*-12 oral administration on intestinal microbiota at the family level of AOM/DSS-treated mice (*n* = 5). (**A**) Bar picture, (**B**) NC vs. AOM/DSS species difference analysis, (**C**) AOM/DSS vs. HLP12 species difference analysis, and (**D**) AOM/DSS vs. 5ASA species difference analysis. * *p* < 0.05.

**Figure 10:**
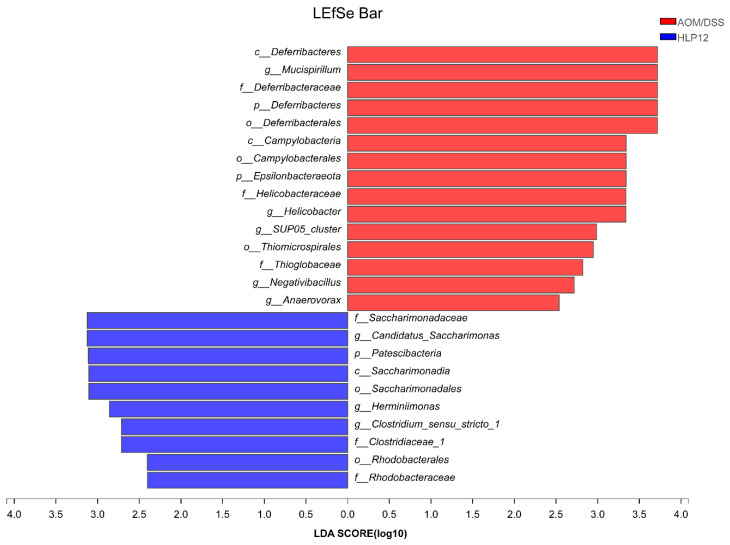
LEfSe analysis of fecal microbiota of AOM/DSS-treated mice (*n* = 5). Biomarker taxa generated from LEfSe analysis (LDA > 2.0) between AOM/DSS and HLP12.

To further investigate specific differences in the intestinal microbiota composition between different groups, we analyzed the microbial community structure profiles of the mice in the four groups. At the phylum level, the bacterial population was primarily composed of *Bacteroidetes*, *Firmicutes*, *Proteobacteria*, *Actinobacteria*, *Verrucomicrobia*, *Patescibacteria*, *Deferribacteres,* and *Epsilonbacteraeota*. Compared to the NC group, the increased content of *Proteobacteria*, *Epsilonbacteraeota, Firmicutes*, and *Deferribacteres* were observed in the AOM/DSS group. However, *L. plantarum*-12 oral administration reversed the trend (Figure 8A–D). *L. plantarum*-12 increased the relative abundance of *Bacteroidetes* in the intestinal microbiota of the AOM/DSS-treated mice. Moreover, at family level, compared to the AOM/DSS group, the contents of beneficial *Muribaculaceae*, *Lactobacillaceae*, *Bifidobacteriaceae*, *Clostridiaceae_1*, and *Saccharimonadaceae* in the intestinal microbiota of the AOM/DSS-treated mice were elevated by *L. plantarum*-12. Meanwhile, the relative abundances of potential pathogenic *Erysipelotrichaceae*, *Helicobacteraceae,* and *Desulfovibrionaceae* in the intestinal microbiota of the AOM/DSS-treated mice were reduced by *L. plantarum*-12 (Figure 9C). In addition, the LEfSe analysis suggested that HLP12 and AOM/DSS groups were characterized by the enrichments in *Saccharimonadaceae* and *Deferribacteraceae* at the family level, respectively (Figure 10).

### 3.7. Effect of L. plantarum-12 oral Administration on Gut Feces Metabolites of the AOM/DSS-Treated C57BL/6 Mice

**Figure 11:**
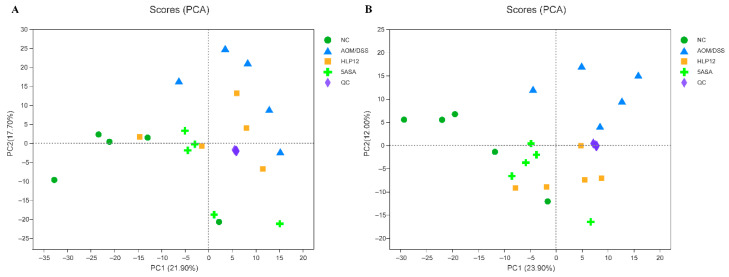
Untargeted fecal metabolomics analysis of AOM/DSS-treated mice (*n* = 5). PCA score plot under negative ion mode (**A**) and positive ion mode (**B**).

**Figure 12:**
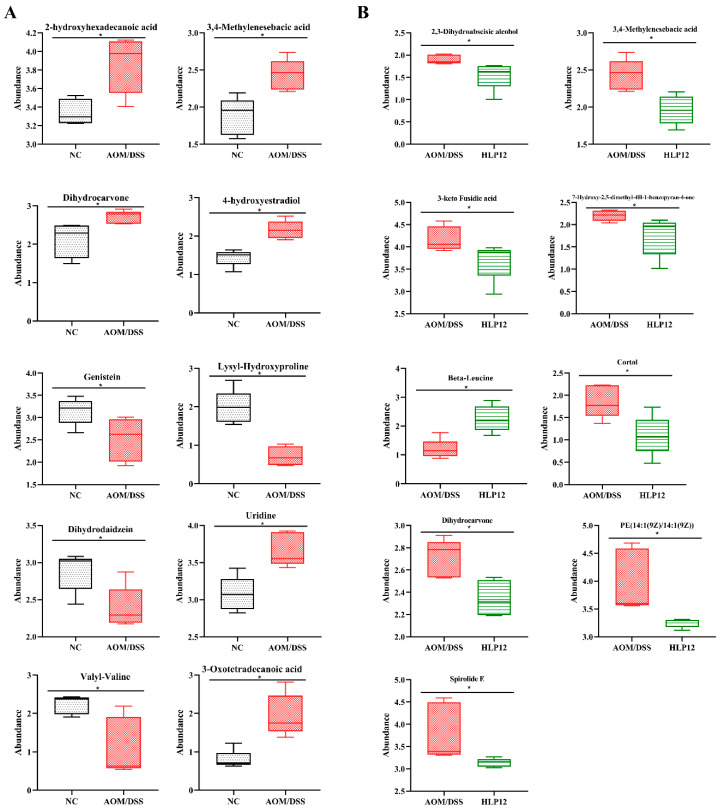
Differential metabolites analysis of the AOM/DSS-treated C57BL/6 mice (*n* = 5). (**A**) NC vs. AOM/DSS differential metabolites and (**B**) AOM/DSS vs. HLP12 differential metabolites. * *p* < 0.05.

Given the ameliorating effect of *L. plantarum*-12 oral administration on AOM/DSS treated C57BL/6 mice, we further investigated the feces metabolites using metabolomics. The PCA analysis was performed using data from feces metabolites of four groups. It was found that PCA successfully distinguished metabolic profile between NC and AOM/DSS groups in negative and positive modes (Figure 11A,B), suggesting that changes occurred in the metabolic profiles of the mice in AOM/DSS group comparing to the NC group. Differential metabolites in feces between AOM/DSS group and NC group can be seen (Figure 12A). Briefly, 2-hydroxyhexadecanoic acid, 3,4-Methylenesebacic acid, 3-Oxotetradecanoic acid, dihydrocarvone, uridine, 4-hydroxyestradiol, etc., were higher in the AOM/DSS group, while genistein, dihydrodaidzein, valyl-valine, lysyl-hydroxyproline, etc., were reduced.

**Figure 13:**
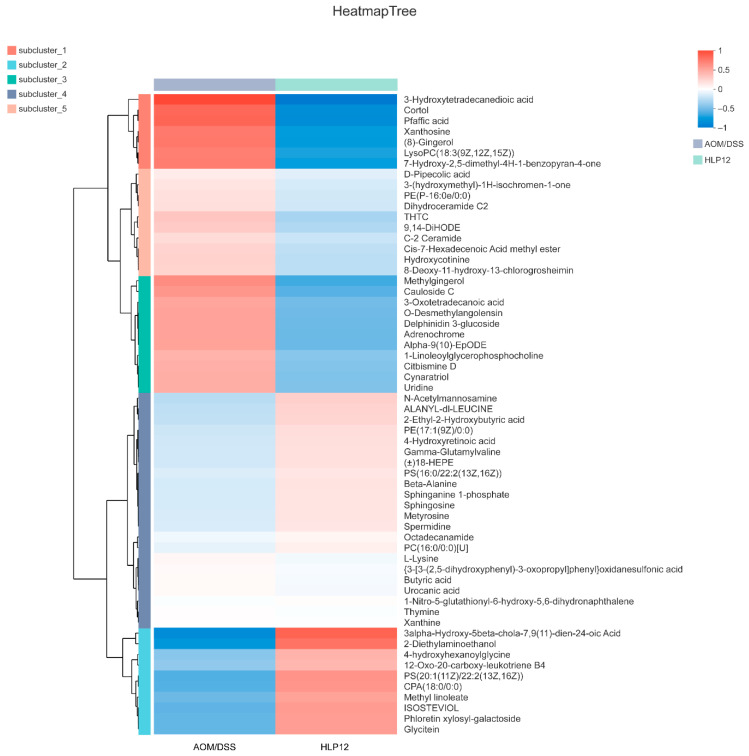
Metabolite cluster analysis of the AOM/DSS-treated C57BL/6 mice in AOM/DSS and HLP12 groups (*n* = 5).

**Figure 14:**
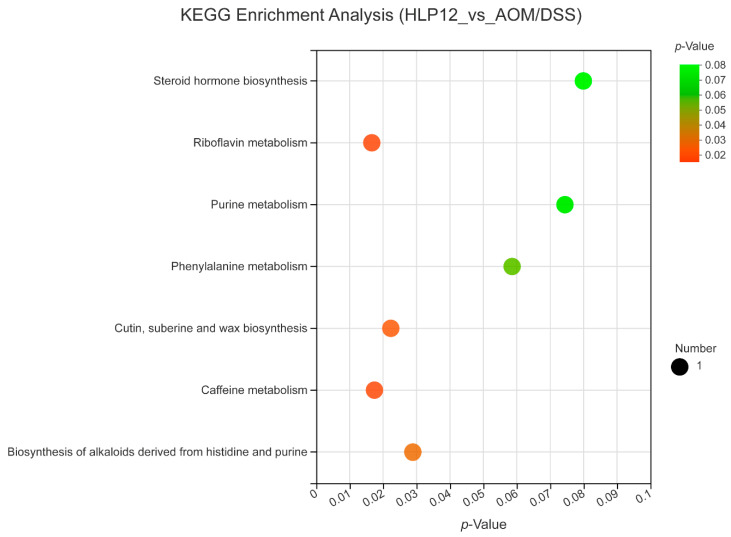
Metabolic pathway enrichment analysis of differentially presented metabolites of the mice in HLP12 and AOM/DSS groups (*n* = 5). Generally, *p* < 0.05 indicates that this function is a significant enrichment item. The size of bubbles represents the amount of metabolites in the pathway.

Furthermore, the 4-hydroxyhexanoylglycine, beta-leucine, phloretin xylosyl-galactoside, and glycitein were significantly enhanced by *L. plantarum*-12 oral administration (Figure 12B and Figure 13). Subsequently, adrenochrome, xanthosine, uridine, 1-linoleoylglycerophosphocholine, LysoPC(18:3(9Z,12Z,15Z)), PE(14:1(9Z)/14:1(9Z)), 3,4-methylenesebacic acid, 3-hydroxytetradecanedioic acid, 3-oxotetradecanoic acid, 7-hydroxy-2,5-dimethyl-4H-1-benzopyran-4-one, 3-keto fusidic acid, and cortol were evidently diminished by *L. plantarum*-12 oral administration compared to AOM/DSS group (Figure 12B and Figure 13). The metabolite pathways analysis suggested that these metabolite pathways were mainly involved in riboflavin metabolism, cutin, suberine, and wax biosynthesis, caffeine metabolism, and biosynthesis of alkaloids derived from histidine and purine (Figure 14).

### 3.8. Correlation Analysis

**Figure 15:**
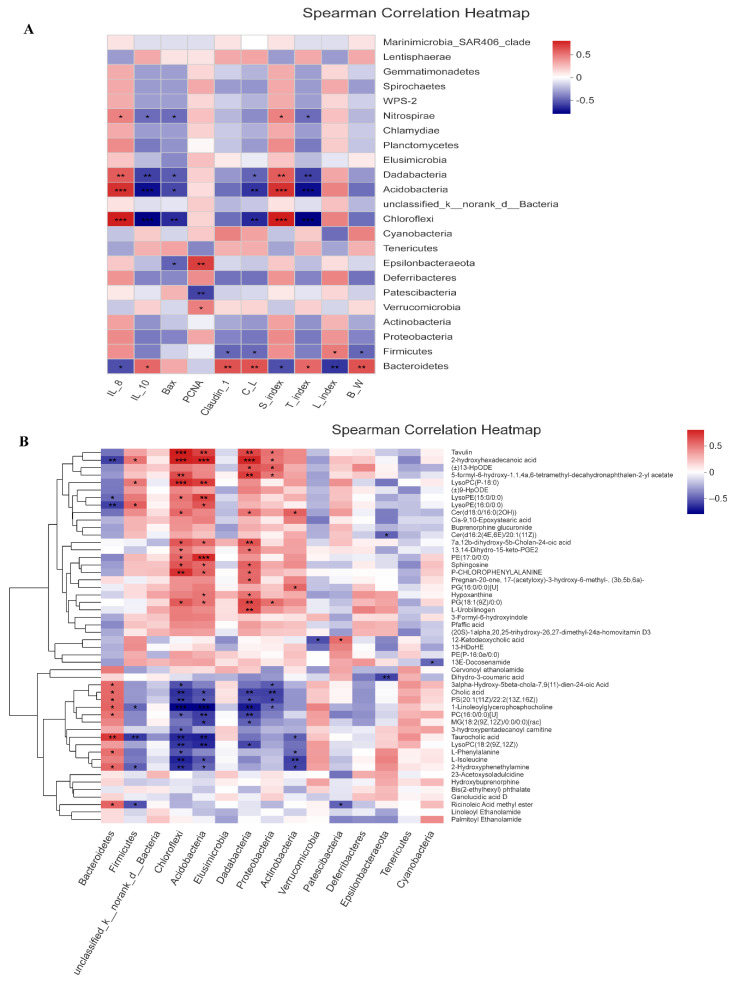
Correlations analyses. (**A**) Bacteria at phylum level were correlated with the phenotypic parameters (*n* = 4). (**B**) Bacteria at phylum level were correlated with the metabolites (*n* = 4). C_L, colon length; S_index, spleen index; T_index, thymus index; L_index, liver index; B_W, body weight. *R* values for the correlation are depicted from red to blue, representing positive and negative correlations, respectively. * 0.01 < *p* ≤ 0.05, ** 0.001 < *p* ≤ 0.01, *** *p* ≤ 0.001.

We next investigated whether the changes in the intestinal microbiota were correlated with the gut inflammation symptoms and the relevance between microflora and the phenotypic parameters of the AOM/DSS-treated mice in all groups (Figure 15A,B). At the phylum level, the increased abundance of *Chloroflexi* had significant positive correlation with the expression of intestinal pro-inflammatory cytokines IL-8, whereas increased level of IL-10 had a significant negative relationship with *Chloroflexi*. Meanwhile, *Epsilonbacteraeota* exhibited a negative correlation with the Bax expression. Furthermore, the abundance of *Firmicutes* was positively correlated with liver index but negatively correlated with body weight, colon length, and claudin-1, respectively. In addition, *Bacteroidetes* showed negative correlation with IL-8, spleen index, and liver index; however, it had positive correlation with IL-10, claudin-1, colon length, thymus index, and body weight, respectively.

Subsequently, we further analyzed the correlation between intestinal microbiota and their metabolites in AOM/DSS-treated mice. The resulting metabolic association heatmap reveals positive and negative correlation between the identified bacterial taxa and the metabolite levels (Figure 15B). *Chloroflexi* showed positive correlations with sphingosine, 2-hydroxyhexadecanoic acid, and cer(d18:0/16:0(2OH)) and negative correlations with l-isoleucine and l-phenylalanine. In addition, *Bacteroidetes* was negatively correlated with LysoPE(16:0/0:0), LysoPE(15:0/0:0), and 2-hydroxyhexadecanoic acid but positively correlated with taurocholic acid and l-phenylalanine. However, *Firmicutes* had significant positive correlation with LysoPE(16:0/0:0), LysoPC(P-18:0), and 2-hydroxyhexadecanoic acid, respectively.

## 4. Discussion

In this study, we constructed an AOM/DSS-treated colon cancer model in C57BL/6 mice to explore the ameliorating effect of *L. plantarum*-12 oral administration on the colon cancer of the mice. The results showed that *L. plantarum*-12 oral administration could modulate the intestinal microbiota, enhance intestinal barrier, alleviate intestinal inflammation, and reduce the tumor burden in AOM/DSS-treated C57BL/6 mice.

The intestinal microbiota is considered as a vital organ in human body. Gut bacterial dysbiosis of host may lead to chronic colonic and systemic inflammation. Accumulating evidence suggests that dysbiosis of the intestinal microbiota has a vital role in the development of intestine diseases, such as inflammatory bowel disease (IBD), irritable bowel syndrome (IBS), and colon cancer [22]. Through high-throughput sequencing technology, we know that the C57BL/6 mice intestinal microbiota is mainly composed of seven phylum, namely *Bacteroidetes*, *Firmicutes*, *Proteobacteria*, *Actinobacteria*, *Verrucomicrobia*, *Patescibacteria*, and *Deferribacteres*. The typical characteristics of intestinal microbiota dysbiosis consist of a decrease in microbiota diversity [23] and in the abundance of some beneficial bacteria such as *Muribaculaceae*, which are short-chain fatty acid (SCFA)-producing bacteria [24], and subsequently, an increased abundance of potentially pathogenic bacteria, such as *Desulfovibrionaceae* is also observed [25]. Our study demonstrated that *L. plantarum*-12 oral administration led to change in intestinal microbiota composition in phylum level, with a decreased abundance of endotoxin-producing *Proteobacteria*. The *Proteobacteria* increase in the colon resulted in inflammation [26]. *Bifidobacterium bifidum* FSDJN7O5 was reported to significantly decrease the relative abundance of *Proteobacteria* in the IBS-diarrhea mice [27]. The supplementation of *L. acidophilus* cell lysates (combined with an anti-CTL antigen-4-blocking antibody) obviously decreased the relative abundance of *Proteobacteria* in the AOM/DSS-treated mice model [28]. Meanwhile, *L. plantarum*-12 oral administration reduced the relative abundance of *Firmicutes* and increased the relative abundance of *Bacteroidetes* in the AOM/DSS-treated mice. Similarly, an increase in the ratio of *Bacteroidetes/Firmicutes* had a preventative effect on colon cancer development [29].

The analysis of the intestinal microbiota of the mice in family levels showed *L. plantarum*-12 oral administration also increased the SCFAs-producing bacteria such as *Muribaculaceae* and *Clostridiaceae_1*. It is well-known that SCFAs play a protective role in colon cancer through various mechanisms, including regulating T-cell homeostasis and epigenetic modification of tumors cells by inhibiting histone deacetylase [30,31]. Moreover, *L. plantarum*-12 oral administration increased the relative abundance of other beneficial microbes such as *Lactobacillaceae* and *Bifidobacteriaceae*. Some species of *Lactobacillaceae* were thought to be beneficial to alleviate several intestine diseases, including IBD [32], IBS [33], and colon cancer [34]. There are compelling data indicating that *Bifidobacteriaceae* exhibited an enhancing effect on epithelial barrier function and short-chain fatty metabolites [35] as well as its critical role in controlling the cancer response to immunotherapy [36]. *L. plantarum*-12 oral administration decreased the level of *Desulfovibrionaceae*, *Erysipelotrichaceae*, and *Helicobacteraceae*, which were positively correlated to colon cancer [34,37,38]. Previous studies have reported that different bacteria showed different effects on colon cancer development. *Desulfovibrionaceae,* as one of the sulfate-reducing and endotoxin-producing bacteria, could be associated with a risk of colon cancer by promoting oxidation and DNA damage [25,39,40]. Meanwhile, *Erysipelotrichaceae* was reported to promote inflammation in the host intestine [37].

The imbalance of intestinal microbiota results in the changes of gut metabolites. In this study, we analyzed the fecal metabolome, serving as a proxy of the intestinal metabolome, as it largely reflects intestinal physiology. *L. plantarum*-12 oral administration significantly decreased fatty acyls, including 3,4-methylenesebacic acid, 3-hydroxytetradecanedioic acid, and 3-oxotetradecanoic acid, in the feces of AOM/DSS-treated C57BL/6 mice. Studies have shown that 3,4-methylenesebacic acid is associated with the development of colon cancer [41]. Similarly, Chen et al. reported that most of the fatty acyls were increased in type 2 diabetic rats induced by high-fat diet [42]. Meanwhile, *L. plantarum*-12 oral administration decreased glycerophospholipids, including PE(14:1(9Z)/14:1(9Z)), LysoPC(18:3(9Z,12Z,15Z)), and 1-linoleoylglycerophosphocholine, in the feces of AOM/DSS-treated C57BL/6 mice. Glycerophospholipids are main lipid components of cell membranes and have extensive functions in cell proliferation, differentiation, and apoptosis [43]. Gao et al. found that the supplement of *L. plantarum* Y44 significantly declined the level of glycerophospholipids in the serum of the D-gal-injection-induced mice, which might alleviate fatty acid oxidation [13], according to reports that nucleosides are potential marker of tumor diagnosis in colon cancer and gastric cancer patients [44,45]. However, fortunately, *L. plantarum*-12 oral administration decreased nucleosides, including xanthosine and uridine, in the feces of AOM/DSS-treated C57BL/6 mice. It is reported that fecal steroids may be related to the etiology of colon cancer [46]. In addition, *L. plantarum*-12 oral administration decreased steroids, including 3-keto fusidic acid and cortol, in the feces of AOM/DSS-treated C57BL/6 mice.

According to our results, *L. plantarum*-12 increased the levels of 4-hydroxyhexanoylglycine and beta-leucine. Hydroxyhexanoylglycine was reported to down-regulate in the kidney cancer samples [47]. Meanwhile, leucine was reported to show lower relative mean intensity value in the colon cancer samples compared to controls [48,49,50]. Furthermore, *L. plantarum*-12 increased the levels of flavonoids, including phloretin xylosyl-galactoside, in the AOM/DSS-treated female C57BL/6 mice. Huo et al. reported that flavonoids prevent colon cancer in an AOM/DSS-treated female C57BL/6 mice model [51]. *L. plantarum*-12 increased the levels of isoflavonoids, including glycitein, in the AOM/DSS-treated mice. A large number of studies have shown that flavonoids and isoflavones have a variety of physiological functions, including anti-proliferation, pro-apoptosis, anti-oxidation, and immune regulation, etc. [52,53,54].

In this study, metabolic pathway enrichment analysis of differentially presented metabolites indicated that oral administration of *L. plantarum*-12 alleviated colon cancer by regulating the pathways involved in riboflavin metabolism, cutin, suberine, and wax biosynthesis, caffeine metabolism, and biosynthesis of alkaloids derived from histidine and purine. The intestinal microbiota changes result in different metabolites in the colon, while the metabolites affect intestinal barrier function and immunity.

The intestinal permeability increased by unfavorable alteration of the gut composition and gut metabolites [13,55]. Therefore, the loss of colon integrity of the AOM/DSS-treated mice was attributed to the intestinal microbiota disturbance. However, *L. plantarum*-12 oral administration improved gut barrier function by increasing tight junction protein Cludin-1. Claudin-1 has been reported to act as vital factor for maintaining intestinal integrity [56,57,58]. AOM/DSS-treated mice exhibited the changes in immune organs, including splenomegaly and thymus atrophy. Meanwhile, *L. plantarum*-12 oral administration alleviated the symptoms of splenomegaly and thymus atrophy in the AOM/DSS-treated mice, indicating the immunoregulating ability of *L. plantarum*-12. Chung et al. reported that treatment with *Aster glehni* obviously attenuated the spleen enlargement of the AOM/DSS-treated colon cancer model mice [59]. Liu et al. reported that tea polysaccharides (TPS) administration significantly increased thymus index in AOM/DSS-treated colon cancer model mice [60].

*L. plantarun*-12 oral administration reduced the tumor burden and increased colon length and body weight in AOM/DSS-treated mice (Figure 2). Accumulating evidence suggests beneficial bacteria and active substances relieve symptoms of colon cancer, including shortening of the colon, tumor burden, and weight loss [23,61,62]. The occurrence and development of tumors were driven by inflammatory immune cells in various occasions, which produced a variety of cytokines that stimulate the tumor cells growth by activating transcription factors such as NF-κB [63,64]. *L. plantarum*-12 oral administration significantly regulated the expression of serum inflammatory factors, evidenced by the reduction of acting factors IL-1β and TNF-α and the increase in the level of anti-inflammatory factor IL-10. Similarly, *L. gasseri* 505 combined with *Cudrania tricuspidata* leaf extract in fermented milk showed a cancer-protective effect on colitis-associated colorectal cancer by down-regulating pro-inflammatory cytokines and up-regulating anti-inflammatory cytokines [65].

It was reported that the NF-κB signaling pathway and MAPK signaling pathway undergo alterations in colon cancer [64,66]. *L. plantarum*-12 oral administration attenuated colon cancer of the AOM/DSS-treated mice via inhibiting NF-κB and p38 MAPK signaling pathways, including down-regulating p65, p-p65, p-p38 and up-regulating IκB-α protein expression. NF-κB activation supports tumorigenesis mainly by increasing colon cancer cell proliferation and angiogenesis, inhibiting colon cancer cell death, and promoting colon cancer cell invasion and metastasis [4]. Our results showed that *L. plantarum*-12 oral administration significantly reduced PCNA protein expression, one of cell proliferation and apoptosis markers, indicating that *L. plantarum*-12 inhibited tumor cell proliferation. Similarly, *Angelica sinensis* root extract administration was reported to depress colonic PCNA expression in the initial colon cancer stage of AOM/DSS-treated mice [67]. Bax, a pro-apoptotic member, works to accelerate programed cell death [68]. *L. plantarum*-12 oral administration significantly increased pro-apoptotic protein Bax level. Similarly, *L. rhamnosus* GG and celecoxib were reported to significantly up-regulate the expression of Bax in the 1,2-dimethylhydrazine-induced colon cancer mice [17]. Collectively, *L. plantarum*-12 oral administration promoted colonic tumor cells death via inhibiting PCNA and increasing pro-apoptotic Bax protein expression.

## 5. Conclusions

This study demonstrated that *L. plantarum*-12 showed significant protective effects against a mice model of colon cancer by regulating the intestinal microbiota and metabolites of AOM/DSS-treated mice, enhancing the intestinal barrier function, and alleviating intestine inflammation. Collectively, *L. plantarum*-12 could be used as a potential probiotic for colon cancer prevention and treatment. In the next study, we will carry out the whole genome analysis of *L. plantarum*-12. It is hoped that *L. plantarum*-12 will be applied to preclinical trials as soon as possible.

## Data Availability

The data presented in this study are available on request from the author.

## Supplementary Materials

The following are available online at https://www.mdpi.com/article/10.3390/nu14091916/s1, Figure S1: Original images of Western blots in the study.
