# Supplementary material for: Lactiplantibacillus plantarum-12 Alleviates Inflammation and Colon Cancer Symptoms in AOM/DSS-Treated Mice through Modulating the Intestinal Microbiome and Metabolome
Source: Nutrients. 2022 May 3;14(9):1916. doi: 10.3390/nu14091916 (PMC9100115; doi:10.3390/nu14091916)
Supplement: Supplementary file 1 [file nutrients-14-01916-s001.zip › nutrients-1635126-supplementary materials.pdf]

Supplementary materials

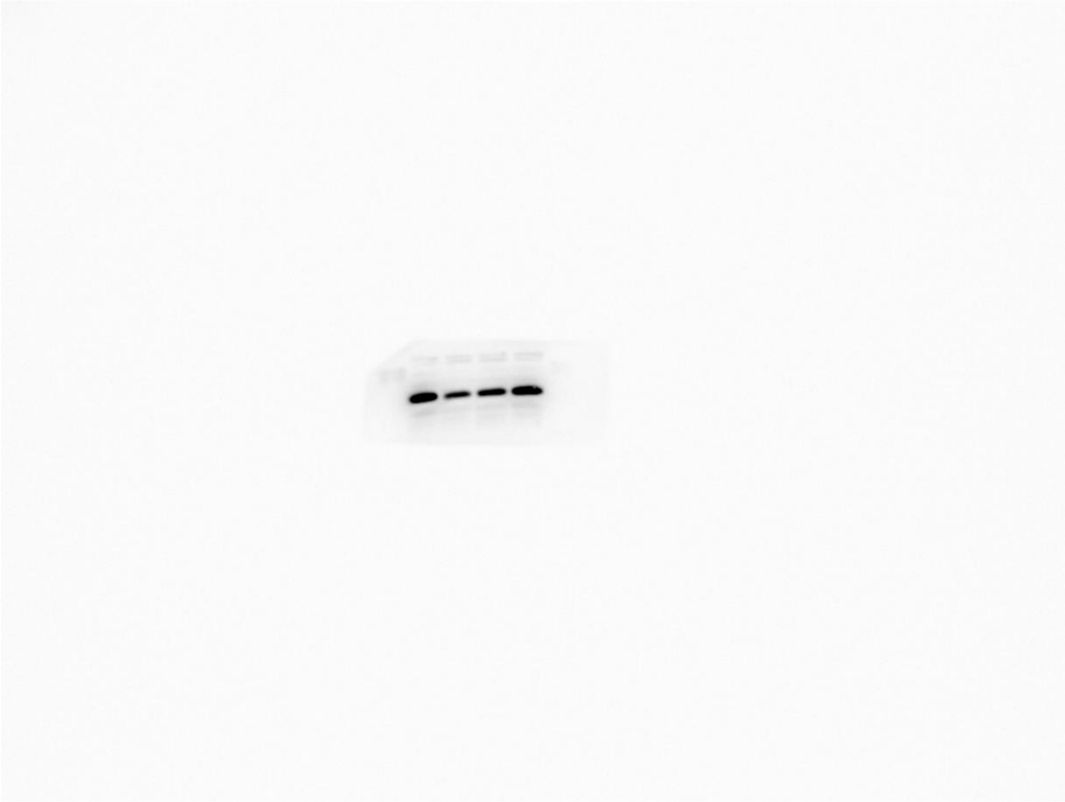

Claudin-1

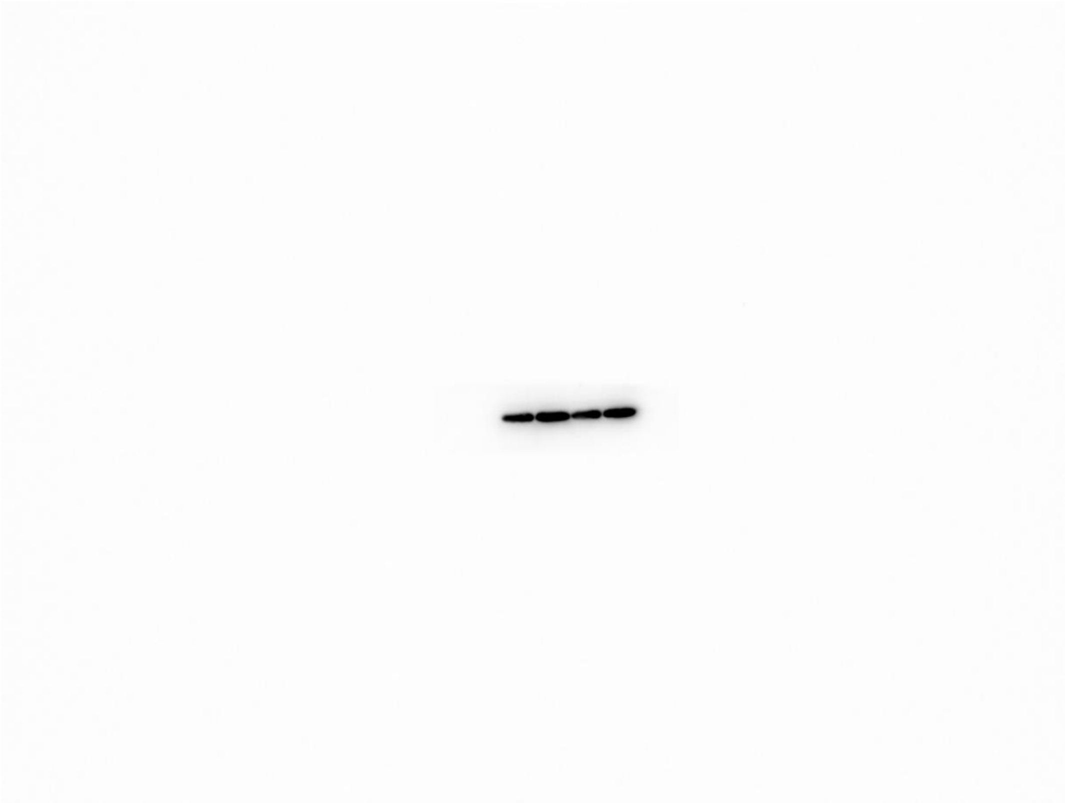

β-actin

Figure 4B

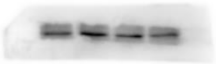

p65

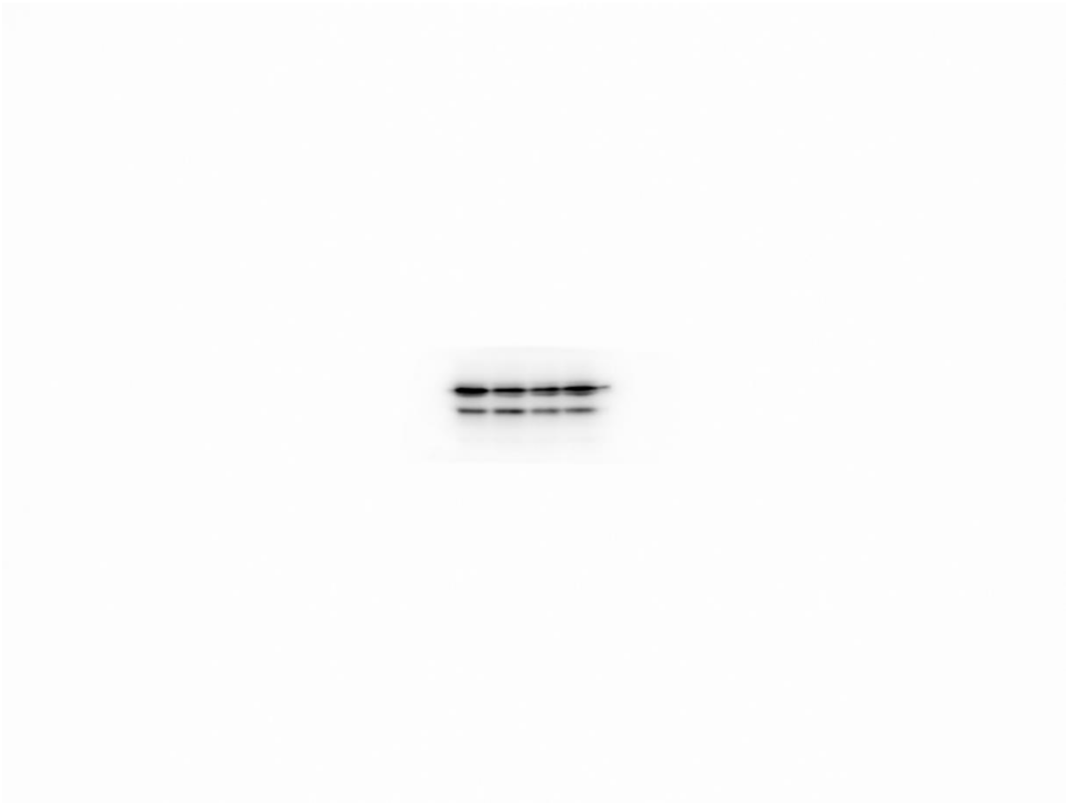

IκB-α

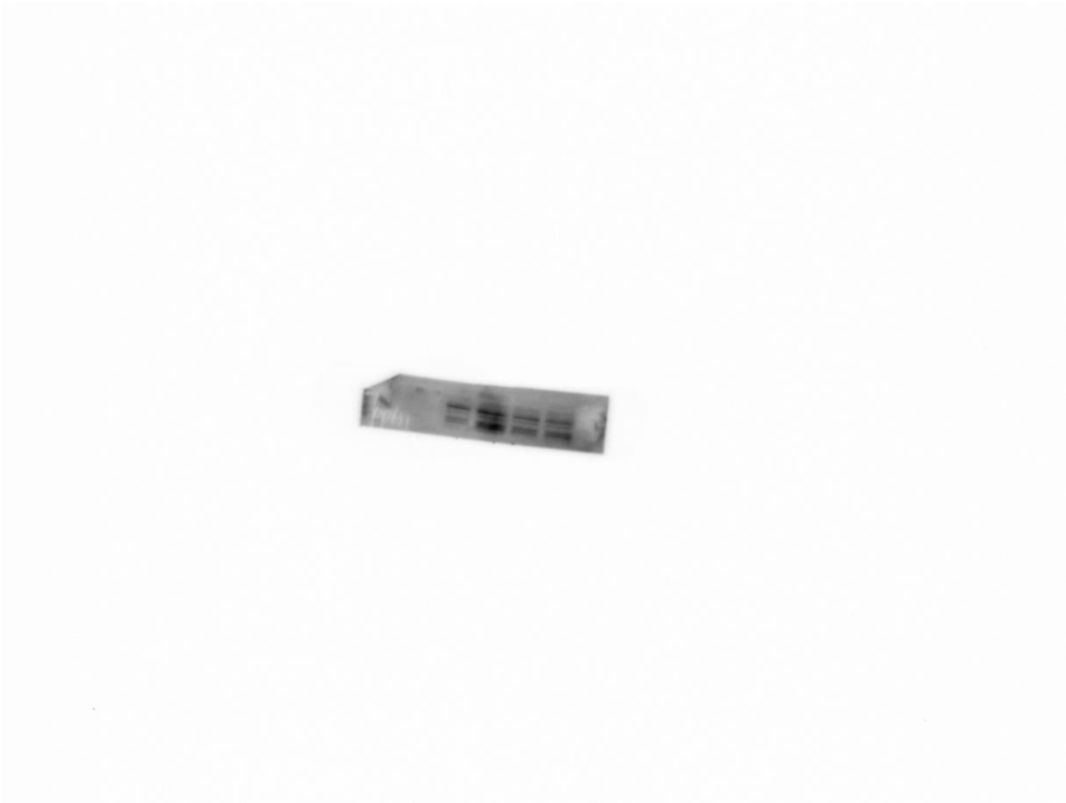

p-p65

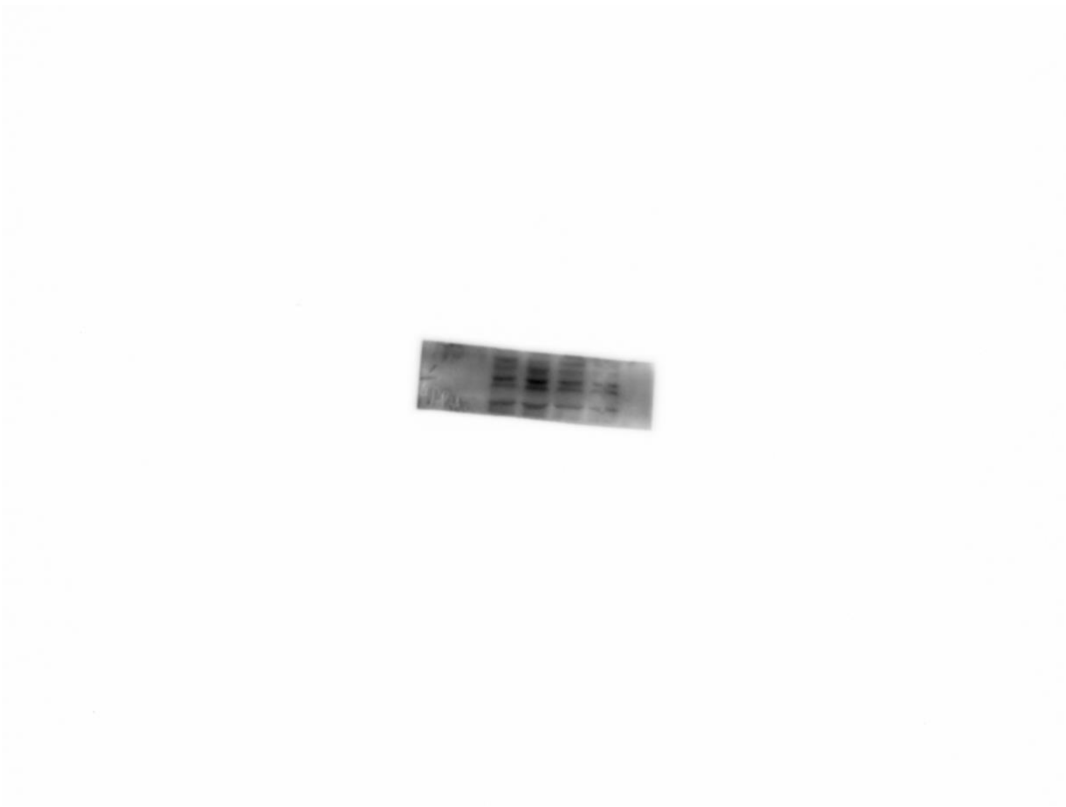

p-p38

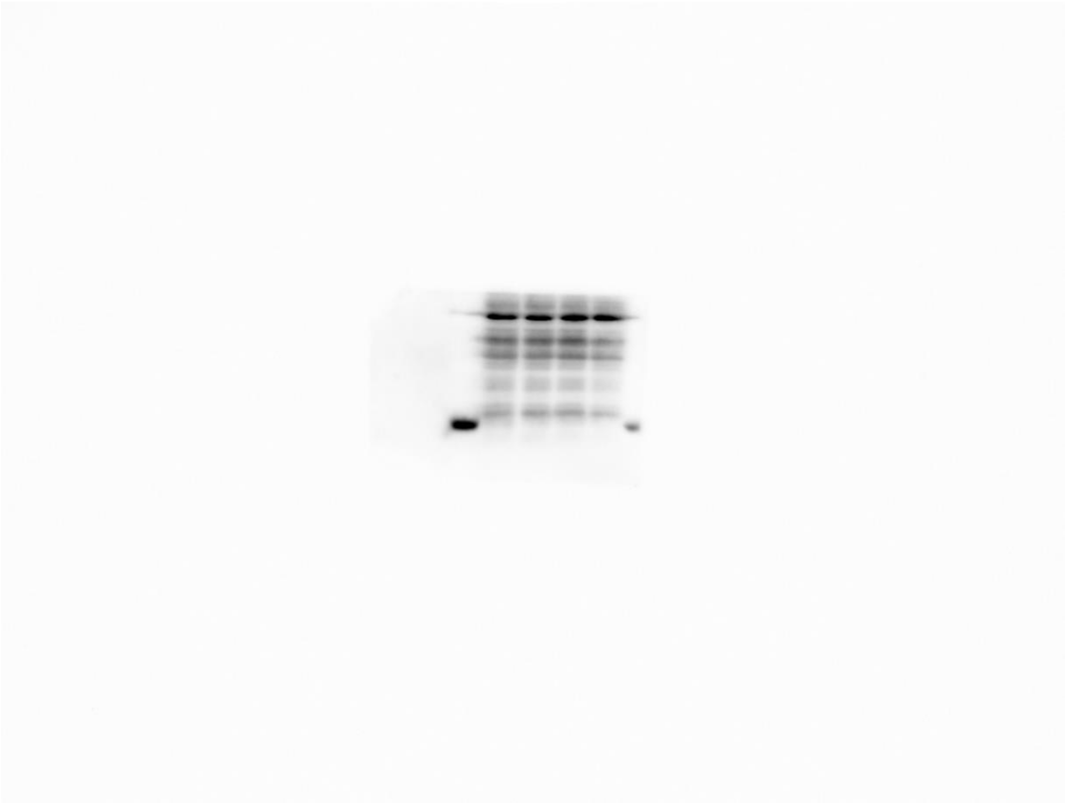

β-actin

Figure 5A

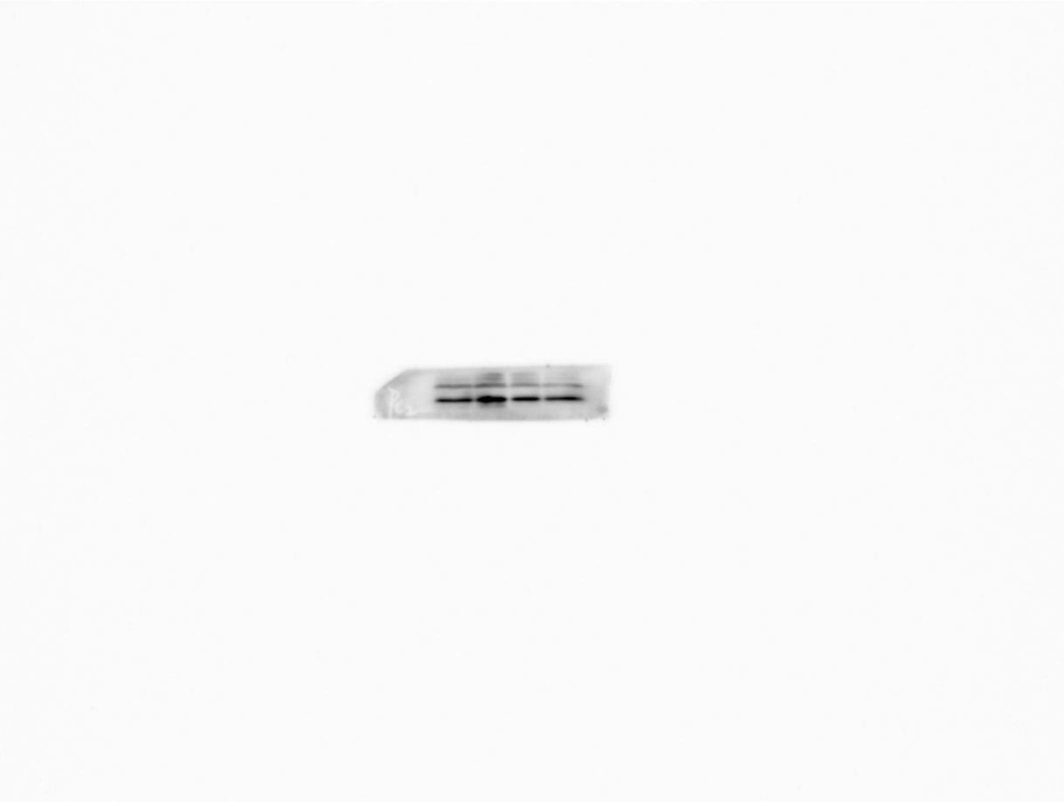

PCNA

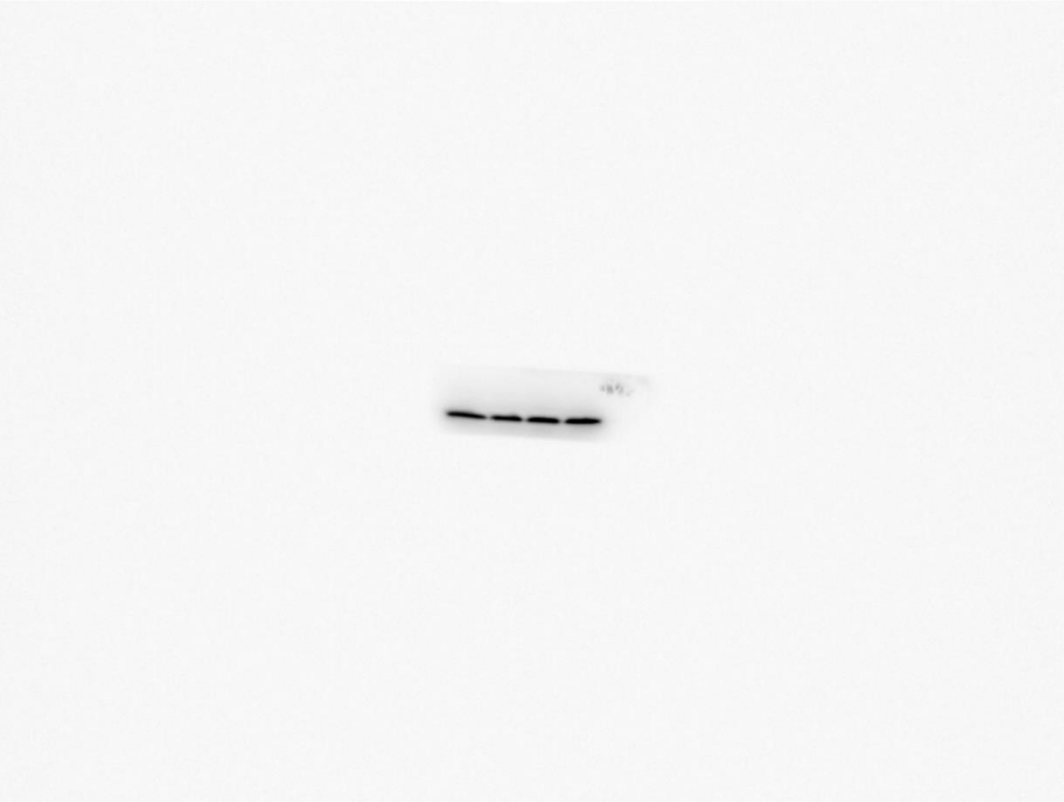

Bax

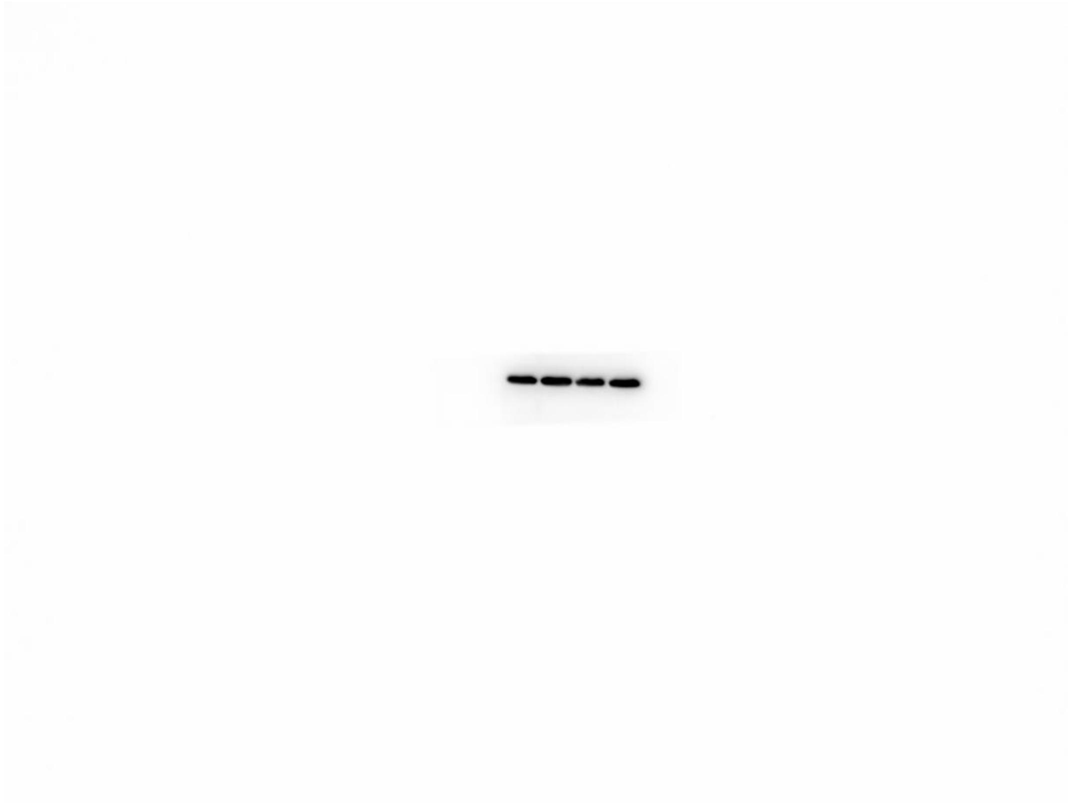

β-actin

Figure 6A

Figure S1. Original images of Western blots.
